# Revealing the Patient Perspective: Evolution of Patient‐Reported Outcome Measures in Botulinum Toxin Studies in Aesthetic Medicine

**DOI:** 10.1111/jocd.70311

**Published:** 2025-06-23

**Authors:** Steven Dayan, Alexander Z. Rivkin, Patricia Ogilvie, Jean D. A. Carruthers, Yan Wu, Elisabeth Lee, Vaishali Patel, Maria Musumeci

**Affiliations:** ^1^ Division of Facial Plastic and Reconstructive Surgery, Department of Otolaryngology University of Illinois at Chicago Chicago Illinois USA; ^2^ DeNova Research Chicago Illinois USA; ^3^ David Geffen/UCLA School of Medicine University of California at Los Angeles Los Angeles California USA; ^4^ Private Practice, Skin Concept Munich Germany; ^5^ Department of Ophthalmology University of British Columbia Vancouver British Columbia Canada; ^6^ Peking University First Hospital Beijing China; ^7^ Clinical Development Head for Toxins, Allergan Aesthetics, an AbbVie Company Irvine California USA; ^8^ Patient Centered Outcomes Research, Allergan Aesthetics, an AbbVie Company Irvine California USA; ^9^ Global Lead Neurotoxin, Global Aesthetics Medical Affairs, Allergan Aesthetics, an AbbVie Company Rome Italy

**Keywords:** botulinum neurotoxin type A, forehead lines, glabellar lines, lateral canthal lines, onabotulinumtoxinA, patient‐focused drug development, patient‐reported outcomes

## Abstract

**Background:**

OnabotulinumtoxinA has been licensed for the treatment of upper facial lines for over 20 years. Patient benefits extend well beyond correction of the lines themselves—including positive effects on confidence, self‐esteem, feelings of attractiveness, and age appearance. Regulatory bodies recommend that patient‐reported outcomes (PROs) are routinely included in registrational studies to ensure the patient perspective is considered. Essential guidance is available from regulators and other expert groups to aid the development, validation, and use of appropriate PROs.

**Aims:**

To reflect on the evolution of patient experience data collection in onabotulinumtoxinA aesthetic medicine development programs within the context of PRO usage across approved botulinum toxins.

**Methods:**

Literature searches were performed to identify relevant publications.

**Results:**

Several PRO instruments have been rigorously developed and validated following regulatory guidance and implemented in onabotulinumtoxinA clinical programs. These include the Facial Line Outcomes Questionnaire (FLO‐11) and Facial Line Satisfaction Questionnaire (FLSQ). The data generated have supported the licensing process and enabled a deeper understanding of patient perspectives. Although these instruments were developed for clinical studies, the concepts obtained directly from patient interviews align with patient goals in clinical practice. Similarly, PROs such as FACE‐Q are now employed more widely across approved botulinum toxins.

**Conclusions:**

A patient‐centric approach, in lockstep with regulatory requirements, has been central to onabotulinumtoxinA development. Awareness of the comprehensive benefits of aesthetic treatments should continue to improve based on increasingly robust PROs.

## 1 Introduction

Botulinum neurotoxin type A (BoNTA) is the most commonly used treatment in aesthetic medicine worldwide [1, 2]. OnabotulinumtoxinA (BOTOX/BOTOX Cosmetic/Vistabel/Vistabex/BOTOX Vista, Allergan Aesthetics, an AbbVie Company, Irvine, CA) was the first BoNTA licensed in this setting and remains the most widely injected and broadly studied product available for treating the upper face—in particular, glabellar (GL), lateral canthal (LCL), and forehead lines (FHL).

The initial licensing of onabotulinumtoxinA for use in aesthetic medicine in 2001 was based on two randomized controlled studies demonstrating improvements in investigator‐assessed GL severity [3, 4]. At that time, there was limited input from patients, and no validated measures of patient satisfaction were available.

Since then, patient‐reported outcomes (PROs) have become an increasingly important element of BoNTA studies in aesthetic medicine—evolving from single‐item scales to score improvements in wrinkle severity to longer multidimensional tools enabling the assessment of emotional and psychosocial effects (Table 1). These newer instruments facilitate a more comprehensive, patient‐centric evaluation of treatment benefit and provide key patient perspective data that can inform clinical practice. Furthermore, the PROs used in clinical studies align closely with the “real world” because they resonate with the goals that patients typically have for their own treatment. They can also support the identification of key unmet treatment needs, thus facilitating the development of new therapies.

**TABLE 1:** Evolution of patient‐reported outcomes assessed in pivotal US and European studies of onabotulinumtoxinA in facial aesthetic indications.

	Indication		
	Glabellar lines	Lateral canthal lines	Forehead lines
Approvals	US: 2002 Europe: 2003	US: 2013 Europe: 2013	US: 2017 Europe: 2017
Phase 3 program	Two DBPC single‐treatment studies with 4 months of follow‐up [3, 4] One open‐label extension study with repeat treatment and 8 months of follow‐up [5]	One DBPC single‐treatment study with 5 months of follow‐up [6] One DBPC repeat‐treatment study with 7 months of follow‐up [7] One DBPC extension study with repeat treatment and 5 months of follow‐up [8]	Two DPBC repeat‐treatment studies with 6 months of follow‐up, and a 6‐month open‐label repeat‐treatment period [9, 10]
Patient‐reported element of the primary endpoint	Global assessment of change in GL appearance	LCL severity at maximum smile using the Facial Wrinkle Scale	FHL severity at maximum eyebrow elevation using the Facial Wrinkle Scale
Other PROs assessing improvements in UFL	*N/A*	LCL severity at rest using the Facial Wrinkle Scale Global assessment of change in LCL appearance	FHL severity at rest using the Facial Wrinkle Scale Global assessment of change in FHL appearance
PROs assessing patient satisfaction	*N/A*	Subject Assessment of Satisfaction of Appearance	Facial Line Satisfaction Questionnaire
PROs assessing psychological/psychosocial benefits	*N/A*	Facial Line Outcomes Questionnaire Self‐Perception of Age	Facial Line Satisfaction Questionnaire Facial Line Outcomes Questionnaire Self‐Perception of Age

Abbreviations: DBPC, double‐blind placebo‐controlled; FHL, forehead lines; GL, glabellar lines; LCL, lateral canthal lines; N/A, not applicable; PRO, patient‐reported outcome; UFL, upper facial lines.

From a regulatory standpoint, a PRO is defined as any measurement of health status that comes directly from the patient themselves without interpretation by another individual [11]. With increasing emphasis on “patient‐focused drug development” (PFDD), PROs are now a key recommendation of regulators worldwide, with published guidance from the US Food and Drug Administration (FDA), European Medicines Agency (EMA), and Chinese Center for Drug Evaluation (CDE).

When the onabotulinumtoxinA clinical programs in LCL and FHL were initiated, there were no existing indication‐specific PROs that met all elements of the FDA guidance. Several instruments were therefore developed and validated for use in these programs, in line with regulatory guidance, including the Facial Line Outcomes Questionnaire (FLO‐11, Copyright Allergan Inc.) and the Facial Line Satisfaction Questionnaire (FLSQ; Copyright Allergan Inc.) [12, 13, 14]. As a result, PRO data in these studies were analyzed with the same scientific rigor as other primary efficacy and safety outcomes.

This review assesses the evolution of PRO measures used across BoNTA upper facial line (UFL) registrational studies through the past, present, and into the future. It has several aims: (1) to describe how the PRO instruments used in onabotulinumtoxinA programs have been developed over the past 20 years in alignment with key regulatory guidance, and assess the broader impact on aesthetic medicine; (2) to set this into context by reviewing PRO measures used across approved BoNTA studies; and (3) to consider likely future developments in this field.

## 2 Methods

To capture all key publications, three separate PubMed database searches were conducted up to and including 19 July 2024 (Table 2). These gave a total of 1239 non‐unique hits. Additional checks for relevant publications were made based on two recent systematic reviews of PRO use with aesthetic BoNTA treatments [15, 16], as well as the personal knowledge of the author group.

**TABLE 2:** Literature search.

Search terms	Limits
(OnabotulinumtoxinA OR abobotulinumtoxinA OR incobotulinumtoxinA OR daxibotulinumtoxinA OR prabotulinumtoxinA OR letibotulinumtoxinA OR botulinum) AND (“patient reported outcome” OR satisfaction OR “psychological impact” OR “quality of life”)	Date (up to and including 19 July 2024) Species (human subjects) Language (English) Article type (“Clinical trial” or “Clinical study”)
OnabotulinumtoxinA AND (“patient reported outcome” OR satisfaction OR “psychological impact” OR “quality of life”) AND (aesthetic OR cosmetic OR lines OR rhytids)	Date (up to and including 19 July 2024)
(AbobotulinumtoxinA OR incobotulinumtoxinA OR daxibotulinumtoxinA OR prabotulinumtoxinA OR letibotulinumtoxinA) AND (“patient reported outcome” OR satisfaction OR “psychological impact” OR “quality of life”)	Date (up to and including 19 July 2024)

Records were then assessed for eligibility: (1) prospective study using PRO instrument(s) to directly evaluate BoNTA outcomes (e.g., satisfaction, goal attainment, naturalness, confidence, emotional impact, or other psychological/psychosocial benefits) in the aesthetic treatment of adults with GL, LCL, and/or FHL; and (2) treatment based on one or more of onabotulinumtoxinA, abobotulinumtoxinA, incobotulinumtoxinA, daxibotulinumtoxinA, prabotulinumtoxinA, or letibotulinumtoxinA. Records were excluded if the study only assessed subject‐reported wrinkle severity appearance.

A total of 102 published papers were identified (onabotulinumtoxinA, *n* = 47; abobotulinumtoxinA, *n* = 28; prabotulinumtoxinA, *n* = 11; incobotulinumtoxinA, *n* = 9; daxibotulinumtoxinA, *n* = 3; letibotulinumtoxinA, *n* = 3; multiple BoNTAs, *n* = 1) (listed in full in the Data S1). All were considered for their relevance to the present review, with the main focus on 38 reports from Phase 3 (registrational) studies: 19 primarily assessing onabotulinumtoxinA [6, 7, 8, 9, 10, 17, 18, 19, 20, 21, 22, 23, 24, 25, 26, 27, 28, 29, 30], seven on prabotulinumtoxinA [31, 32, 33, 34, 35, 36, 37], six on abobotulinumtoxinA [38, 39, 40, 41, 42, 43], three on letibotulinumtoxinA [44, 45, 46], two on daxibotulinumtoxinA [47, 48], and one on incobotulinumtoxinA [49].

## 3 OnabotulinumtoxinA Indication in GL: Reliance Largely on the Clinician Perspective

**FIGURE 1:**
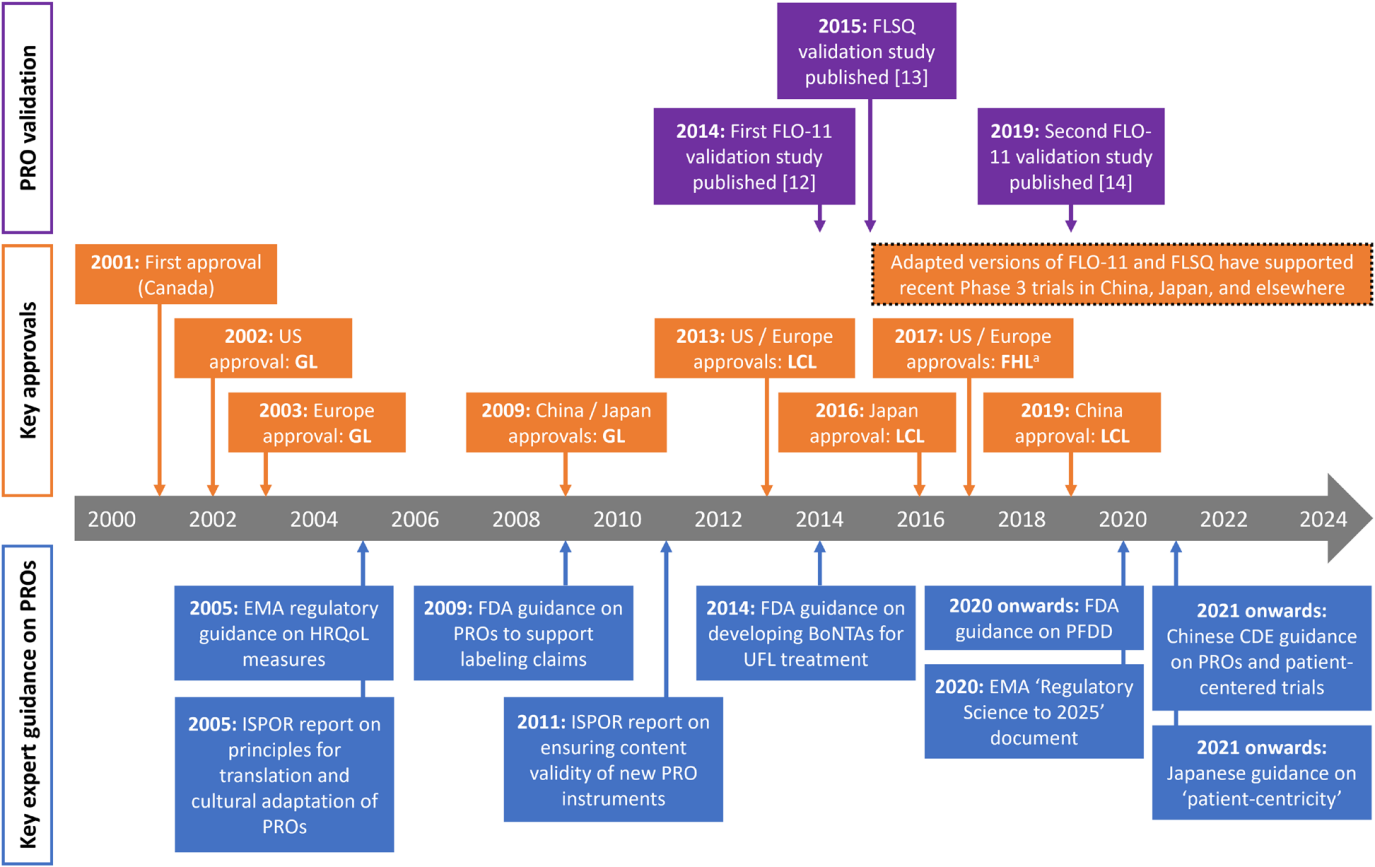
Timeline of key developments: PROs and onabotulinumtoxinA in facial aesthetic indications. ^a^The clinical program underlying these approvals incorporated patients treated in all three upper facial areas (GL, LCL and FHL). CDE, Center for Drug Evaluation; EMA, European Medicines Agency; FDA, Food and Drug Administration; FHL, forehead lines; FLO‐11, Facial Line Outcomes Questionnaire; FLSQ, Facial Line Satisfaction Questionnaire; GL, glabellar lines; HRQoL, health‐related quality of life; ISPOR, Professional Society for Health Economics and Outcomes Research; LCL, lateral canthal lines; PFDD, patient‐focused drug development; PRO, patient‐reported outcome; UFL, upper facial lines.

Two multicenter, randomized controlled studies conducted in 1999 established the efficacy and safety of onabotulinumtoxinA treatment of moderate‐to‐severe GL (Table 1) [3, 4]. Both showed that onabotulinumtoxinA significantly decreased investigator‐rated GL severity compared with placebo, using the clinician‐assessed Facial Wrinkle Scale (FWS). This led to initial approval in Canada in 2001, followed by the US in 2002, and Europe in 2003 (Figure 1).

At that time, there was limited regulatory guidance for the development and validation of PROs and no requirement to include them in clinical studies. Thus, the only patient‐rated endpoint in those studies was the Global Assessment of Change. This single‐item tool enabled patients to assess their GL severity using a nine‐point scale but did not consider treatment satisfaction or any psychosocial benefits.

## 4 OnabotulinumtoxinA Indication in LCL: Advancing the Patient Perspective

### 4.1 PRO Measures Within the Program

Pivotal studies conducted in 2010–11 demonstrated the efficacy and safety of onabotulinumtoxinA for the treatment of moderate‐to‐severe LCL [6, 7, 8], leading to approval in the US and Europe in 2013. Following FDA feedback, clinician‐ and subject‐reported LCL severity were included in these trials as a composite primary endpoint [6, 7].

The LCL clinical program was the first to incorporate other PRO instruments going beyond facial line severity, demonstrating that onabotulinumtoxinA treatment was associated with significant improvements in patient perceptions of their overall facial appearance (e.g., attractiveness, tiredness, age) and greater satisfaction with results compared with placebo [6, 7, 8, 17]. These studies also assessed the benefits of simultaneous injection of LCL and GL versus LCL treatment alone, and found greater improvements in PRO measures with the combination approach [7, 8, 17].

Analyses were based on FLO‐11, Self‐Perception of Age (SPA), and the Subject Assessment of Satisfaction of Appearance (SASA) tool (Table 1). FLO‐11 encompassed a broad group of concepts related to UFL, allowing patients to assess how they look (tired, stressed, angry, not having a smooth face, not rested), how they feel (older, less attractive), and how much their facial lines bother them [12, 14]. It was developed and validated for all UFL in accordance with FDA and EMA guidance and was initially implemented in the LCL pivotal trial program, as well as a smaller non‐registrational study of 70 patients with GL [50].

### 4.2 Expert Guidance Feeding Into the Work

In the US, the FDA published their *draft* guidance on PROs in product development in 2006, providing detailed methodology for ensuring the adequacy of PRO instruments to support labeling claims [51]. This guidance outlined an iterative process for identifying concepts and developing a conceptual framework, creating the instrument, assessing measurement properties, and modifying the instrument as needed.

In Europe, the EMA published a “reflection paper” in 2005, giving regulatory guidance on the use of health‐related quality of life (HRQoL) measures for evaluating medicinal products [52]. This document was not intended to provide specific requirements for developing and validating PRO measures. Instead, it outlined important principles for capturing HRQoL in drug development studies (e.g., outcome evaluation directly by the patient themselves, usage of multidimensional instruments when appropriate, and validation in the relevant condition).

Another key document underlying the use of PROs in the onabotulinumtoxinA LCL clinical program was a 2005 report by the Professional Society for Health Economics and Outcomes Research (ISPOR) on principles of good practice for the translation and cultural adaptation of PRO instruments [53]. Using this framework, FLO‐11 has now been translated into more than 10 languages, with specific versions for each type of UFL.

### 4.3 Impact on the Field of Aesthetic Medicine

The novel PROs used in the onabotulinumtoxinA LCL clinical program yielded substantial new insights about patients' treatment experience. In particular, FLO‐11 data highlighted the patient‐perceived emotional and psychosocial benefits—including significant improvements in perceptions of their overall facial appearance with regard to attractiveness, tiredness, age, etc. The LCL studies modeled how to incorporate PROs into clinical development programs as a mechanism for demonstrating the wider treatment benefits beyond an anatomical focus on facial line severity.

## 5 OnabotulinumtoxinA Indication in FHL: Comprehensive Capture of the Patient Perspective

### 5.1 PRO Measures Within the Program

Two multicenter, randomized, controlled, repeat‐treatment studies conducted in 2014–16 demonstrated the efficacy and safety of onabotulinumtoxinA treatment of FHL [9, 10, 18, 19, 20]. One of these also assessed treatment of all three upper facial areas (GL, LCL, and FHL), showing significant appearance‐related psychological and emotional improvements using such an approach [10, 20]. The studies supported onabotulinumtoxinA approval for this indication in the US and Europe in 2017.

The composite primary endpoint (for the US FDA) in the two pivotal FHL studies included both investigator and subject assessment of FHL severity using the FWS. This aligned with 2014 FDA guidance on developing BoNTA products for treating UFL, which recommended using both investigator‐ and patient‐assessed indication severity as the primary endpoint [54]. The FHL clinical program broadened the scope of PROs beyond the LCL program, incorporating FLO‐11 and SPA as secondary endpoints, along with the novel FLSQ instrument (Table 1). FLSQ demonstrated that patients in these studies experienced clinically meaningful psychosocial impacts at baseline due to their UFL appearance and revealed significant patient‐reported improvements, as well as high levels of satisfaction with onabotulinumtoxinA treatment [18, 19, 20].

### 5.2 Expert Guidance Feeding Into the Work

By the time of these studies, final FDA guidance on the use of PROs in product development had been published [11]. In addition, in 2011, the ISPOR PRO Good Research Practices Task Force published a two‐part report on ensuring and documenting the content validity of new PRO instruments [55, 56]. These documents laid out specific practices for designing content validity studies, as well as for evaluation and reporting. Key topics included development and documentation of items included in the instrument, coding of qualitative data, cognitive debriefing interviews with patients, and documentation for submission to regulatory authorities.

To meet FDA requirements ahead of using FLO‐11 in the FHL clinical program, further patient research was conducted, in alignment with ISPOR guidance [12, 14]. Additional qualitative data was collected to support FLO‐11 content validity for UFL; assessing whether it could fully and precisely capture patients' perspectives on the impact of these lines; and evaluating its understandability and ease‐of‐use by patients.

At the same time, the FLSQ was developed, validated, and implemented in the FHL clinical studies in accordance with FDA, EMA, and ISPOR guidance. FLSQ incorporates an even broader set of concepts than FLO‐11 for exploring patient perspectives. It has baseline and follow‐up versions, and assesses various aspects relating to treatment expectations and satisfaction (e.g., onset, duration, improvement in facial lines, natural look), emotional/psychosocial impact (e.g., feeling old/angry/tired, effects on self‐esteem), likelihood of continuing treatment or recommending it to a friend, and the degree to which expectations were met [13]. Patient input was integrated throughout the development process. This included concept elicitation interviews to understand the impact of UFL and expectations around treatment before generating draft questionnaires; cognitive interviews to assess clarity and comprehensiveness and remove redundant items; and psychometric analyses to assess the reliability and validity of the final tool [13].

Large numbers of patients were involved in these programs. For FLO‐11, the development and validation studies conducted in 2008–14 included 98 and 1012 individuals, respectively; for FLSQ, the development and validation work carried out in 2013–16 incorporated 76 and 671 individuals, respectively.

### 5.3 Impact on the Field of Aesthetic Medicine

The onabotulinumtoxinA FHL clinical program was crucial in demonstrating that treatment of all UFL areas is not only efficacious and safe but also has profound psychosocial benefits. In addition, the layers of this benefit were further clarified with the introduction of the FLSQ tool, which incorporated wider concepts, as described above. FLSQ results from onabotulinumtoxinA studies can now form the basis for clinician–patient dialogue regarding the improvements that can be expected from treatment.

Furthermore, because FDA guidance was followed, PRO (FLSQ) data from this onabotulinumtoxinA study program were—for the first time—included within the US Prescribing Information for a BoNTA product [57]. Results from FLSQ and FLO‐11 are also described in detail in the European Summary of Product Characteristics [58], and PRO data are included in the onabotulinumtoxinA product labels for some other countries, such as China [59].

## 6 Recent Use of PROs With OnabotulinumtoxinA

PRO instruments, particularly FLSQ and FLO‐11, continue to be a pillar of global onabotulinumtoxinA development programs. For example, three Phase 3 studies conducted in China and Japan deployed appropriately translated and culturally adapted versions of these questionnaires [21, 22, 23, 24]. The first analyzed 417 Chinese patients randomized to a single treatment with onabotulinumtoxinA or placebo for LCL [21, 22]. The second assessed 300 Japanese patients receiving up to five cycles of onabotulinumtoxinA treatment for LCL (two cycles placebo‐controlled) [23], while the third evaluated 101 Japanese patients receiving up to four cycles of onabotulinumtoxinA for LCL and GL [24]. These studies demonstrated high levels of satisfaction with outcomes, improvements in self‐perception of age, and significant appearance‐related and emotional effects.

In addition, novel PRO instruments—developed following relevant regulatory guidance—are central to the assessment of onabotulinumtoxinA treatment impact in studies of potential new indications in aesthetic medicine, such as platysma prominence [60, 61] and masseter prominence (NCT04073303).

## 7 PRO Usage in Key Studies of Other BoNTAs

Our literature search identified 19 publications from Phase 3 studies of other approved BoNTAs, using PROs to assess UFL treatment (excluding studies limited to subject‐assessed facial lines severity) [31, 32, 33, 34, 35, 36, 37, 38, 39, 40, 41, 42, 43, 44, 45, 46, 47, 48, 49].

Six of these papers reported results from four Phase 3 studies of abobotulinumtoxinA [38, 39, 40, 41, 42, 43]. One found improvements in patients' self‐perception of age over multiple cycles of GL treatment [43]. The other three studies also evaluated GL treatment and all used validated FACE‐Q scales—demonstrating significant improvements in satisfaction with appearance, psychological well‐being, and perception of aging (Table 3) [38, 39, 40, 41, 42]. The FACE‐Q Aesthetics module includes over 40 PRO scales and checklists [62, 63, 64, 65, 66, 67], which can be licensed for use in clinical practice or trial settings.

**TABLE 3:** Multidimensional PRO instruments used in published Phase 3 (registrational) studies.

Botulinum toxin	Instrument	Validated?	Specific to aesthetic medicine?	Specific to patients with facial lines?
OnabotulinumtoxinA [6, 7, 8, 9, 10, 17, 18, 19, 20, 21, 22, 23, 24, 25, 26, 27]	FLO‐11	**✓**	**✓**	**✓**
	FLSQ	**✓**	**✓**	**✓**
AbobotulinumtoxinA [38, 39, 40, 41, 42]	FACE‐Q	**✓**	**✓**	×/**✓** ^a^
LetibotulinumtoxinA [46]	FACE‐Q	**✓**	**✓**	×/**✓** ^a^
	Modified Skindex‐16 GL‐QoL	**✓** (not yet published)	**✓**	**✓**
PrabotulinumtoxinA [32, 33]	HADS	**✓**	×	×
DaxibotulinumtoxinA	N/A	—	—	—
IncobotulinumtoxinA	N/A	—	—	—

Abbreviations: FLO‐11, Facial Line Outcomes Questionnaire; FLSQ, Facial Line Satisfaction Questionnaire; GL, glabellar lines; HADS, Hospital Anxiety and Depression Scale; N/A, not applicable; QoL, quality of life; UFL, upper facial lines.

^a^ Some of the FACE‐Q scales used in these studies are specifically for use in patients with facial lines (e.g., “Appraisal of Lines Between Eyebrows Scale”) while others are not (e.g., “Psychological Well‐being”).

One abobotulinumtoxinA study also employed a 10‐item subject satisfaction questionnaire to analyze domains such as appearing less tired, appearing more youthful, naturalness of the results, and feeling better about themselves [42]; no details were provided regarding the development and validation of this PRO instrument.

For letibotulinumtoxinA, three publications were identified that included PROs [44, 45, 46]. The first two were from Korean studies showing improved patient satisfaction with their facial appearance following treatment of GL or LCL [44, 45]. The third assessed more complex patient‐reported domains using data from three US and European Phase 3 studies of GL treatment [46]. These analyses demonstrated significant improvements with letibotulinumtoxinA versus placebo, based on different PRO instruments—including FACE‐Q (age appraisal and satisfaction with outcomes) and the Modified Skindex‐16 GL Quality of Life Scale. The latter is based on the more general Skindex‐16 tool for measuring the effects of skin disease on quality of life [68]; details of the validation process for the modified instrument were not published.

For prabotulinumtoxinA, there have been seven publications based on four Phase 3 studies that incorporated PROs [31, 32, 33, 34, 35, 36, 37]. The first was a Korean split‐face analysis of the treatment of LCL with prabotulinumtoxinA or onabotulinumtoxinA, which noted similar levels of patient satisfaction between groups and improvements in subjects' age perception [31]. The other three were US and international studies, which demonstrated patient satisfaction with outcomes following treatment of GL with prabotulinumtoxinA [32, 33, 34, 35, 36, 37]. One of these studies also used the validated 14‐item Hospital Anxiety and Depression Scale (HADS) to assess psychological wellbeing [32]. They found significant improvements on HADS with prabotulinumtoxinA at day 90, although there was no clear difference relative to the placebo group at this timepoint.

Finally, with incobotulinumtoxinA and daxibotulinumtoxinA, our searches identified one and two published Phase 3 studies, respectively, that assessed PROs following the treatment of UFL [47, 48, 49]. These evaluations were limited only to patient satisfaction with outcomes, demonstrating positive effects that were consistent with other approved BoNTA treatments for UFL.

## 8 Limitations and Future Directions in PFDD

We should acknowledge the limitations of the present review. As with any literature search, we cannot rule out the possibility that some relevant publications were not captured. In addition, the included studies assessed multiple different BoNTA products and were performed over a long period of time; inevitably, there is heterogeneity in study designs, populations, treatment areas, PRO tools used, and the regulatory backdrop against which individual trials were conducted, which complicates comparison. Nonetheless, we have tried to clarify some of these differences through the chronological perspective employed in this paper. Furthermore, not all of the PRO instruments used were validated according to the same stringent regulatory standards as FLO‐11, FLSQ, and FACE‐Q. A potential limitation of FACE‐Q is that it remains unclear from published literature whether development and validation included robust samples of UFL patients to meet regulatory guidance [63, 69].

Looking forwards, the value of PROs is increasingly viewed within the broader concepts of PFDD and patient experience data (PED). This new direction will be a key driver of future clinical programs in aesthetic medicine. The FDA states that PFDD must employ systematic approaches to ensure patients' experiences, perspectives, needs, and priorities are meaningfully incorporated into product development throughout their lifecycles [70].

A key stipulation of the 2016 “21st Century Cures Act” was that the FDA must develop guidance on data collection and usage relating to PFDD [71]. This commitment was also made within the US Prescription Drug User Fee Act VI, authorized in 2017 [72]. As a result, the FDA has released its PFDD guidance series, with four documents offering detailed information on collection and submission of PED to the FDA during the drug development process (Table 4) [70, 73, 74, 75]. This expands on the 2009 FDA PRO guidance and provides direction on incorporating the patient perspective into drug development processes, sources and methods for collecting PED, and PRO selection, development, or modification.

**TABLE 4:** FDA guidance on data collection and usage relating to patient‐focused drug development.

Guidance	Title	Key topics	Status
1	Collecting Comprehensive and Representative Input [70]	Who to get input from; methods for collecting patient experience data	Published
2	Methods to Identify What Is Important to Patients [73]	Methods for collecting information on areas that are important to patients; best practices for undertaking qualitative research	Published
3	Selecting, Developing, or Modifying Fit‐for‐Purpose Clinical Outcome Assessments [74]	Decision making around what to measure within product development programs; guidance on how to develop and validate fit‐for‐purpose COAs (including PROs)	Draft
4	Incorporating Clinical Outcome Assessments into Endpoints for Regulatory Decision Making [75]	How COAs can be built into relevant endpoints for regulatory use; how to define meaningful changes within those endpoints	Draft

Abbreviations: COA, clinical outcome assessment; FDA, US Food and Drug Administration; PRO, patient‐reported outcome.

Outside of the US, regulatory guidance around PROs and the broader concept of PFDD continues to evolve. For example, the CDE in China has published draft guidelines on PED, the design and implementation of patient‐centered clinical trials, and the use of PROs (Figure 1) [76, 77]. These align with the FDA's PFDD guidance and describe the full scope of the potential application of PROs in drug registration studies; broad principles for their development, translation, and use; considerations on quality control in data collection; and guidance on data analysis and interpretation [76]. Similarly, in Japan, there is an increasing focus on patient‐centricity, and their “Pharmaceuticals and Medical Devices Agency” has set up a working group to develop relevant guidance [78, 79]. Meanwhile, in Europe, the EMA has published its strategic goals up to 2025 [80]. This document reiterates the need for systematic incorporation of PROs into the drug development process and acknowledges the requirement for improved regulatory guidelines around their assessment and application.

## 9 Conclusions

There is increasing awareness among patients, clinicians, and researchers that UFL treatment with BoNTA products can have profound emotional and psychosocial benefits relating to improvements in overall facial appearance—including effects on confidence, self‐esteem, feelings of attractiveness, and age appearance. This may also align with the motivations of younger generations of aesthetic patients, who are increasingly thinking beyond physical beauty towards psychological “wellness.”

A patient‐centric approach—in lockstep with regulatory requirements—has been central to the development of onabotulinumtoxinA in aesthetic indications. Two novel UFL PROs (FLO‐11 and FLSQ) were developed, validated, and implemented into onabotulinumtoxinA pivotal studies in accordance with the rigorous standards set by regulators and other expert groups. The data generated have supported the licensing process and are incorporated into the product label in many countries [57, 58, 59].

The results have enabled a deeper appreciation of the patient perspective, ensuring a more comprehensive assessment of treatment benefits. This is particularly important in aesthetic medicine, where the patient is the fundamental “decision maker.” Moreover, the concepts in these PROs were obtained directly from patients and hence align with goals in clinical practice. PROs are important for assessing the outcomes that ultimately matter most, thereby facilitating physician–patient communication around the potential benefits and informing individualized treatment decision‐making.

The onabotulinumtoxinA trial program has been at the forefront of implementing novel PROs to assess the psychological impact of treatment. In the wider context, such tools are now being incorporated into Phase 3 UFL studies of other BoNTAs. Various instruments, including FACE‐Q, Skindex‐16, and HADS, have been used for this purpose, and the validated FACE‐Q scales are particularly widely applied across aesthetic medicine. However, new indication‐specific PROs may be necessary when existing tools do not fully meet the strict regulatory guidance for labeling—such as development in the same population as the clinical studies, and being fit‐for‐purpose in assessing all relevant concepts for the drug and indication of interest.

The field of aesthetic medicine has an opportunity to take a leading role in the implementation of PFDD across medical product development as a whole. In the future, validated tools should be incorporated more widely into clinical development programs, facilitating the progress of new treatments where there is unmet need. Consideration should also be given to how PROs can be leveraged in clinical practice for active tracking of outcomes in alignment with trials.

## Author Contributions

All authors were involved in the interpretation of data for the work and in drafting and revising the manuscript critically for important intellectual content; gave final approval of the version to be published; and agreed to be accountable for all aspects of the work.

## Ethics Statement

The authors have nothing to report.

## Consent

The authors have nothing to report.

## Conflicts of Interest

Steven Dayan is a clinical investigator and consultant for AbbVie, Galderma, Merz, and Revance. Alexander Rivkin is an investigator, speaker, and consultant for AbbVie, Galderma, Merz, and Suneva. Patricia Ogilvie is an investigator for AbbVie, Galderma, Merz, Meditox, and Evolus, and a consultant for Revance. Jean Carruthers is a consultant and investigator for Acorn, Alastin, Appiell, AbbVie, Avari, Bonti, Del Nova, Evolus, Fount Bio, In Mode, Inverse Genomic, Jeune Aesthetics, Merz, Object Pharma, Revance, and Sofwave. Yan Wu is an investigator for AbbVie. Elisabeth Lee, Vaishali Patel, and Maria Musumeci are employees of Allergan Aesthetics, an AbbVie company.

## Supporting information


Data S1.

## Data Availability

Data sharing is not applicable to this article as no datasets were generated or analyzed.
