# Supplementary material for: Revealing the Patient Perspective: Evolution of Patient‐Reported Outcome Measures in Botulinum Toxin Studies in Aesthetic Medicine
Source: J Cosmet Dermatol. 2025 Jun 23;24(7):e70311. doi: 10.1111/jocd.70311 (PMC12183674; doi:10.1111/jocd.70311)
Supplement: Supplementary file 1 — Data S1. [file JOCD-24-e70311-s001.docx]

**SUPPLEMENTARY FILE: LITERATURE SEARCH RESULTS**

**OnabotulinumtoxinA [47]**

- Dayan S, Ogilvie P, Boyd C, et al. Self-perception of natural outcome, appearance, and emotional well-being after OnabotulinumtoxinA treatment for upper facial lines: Post hoc analysis across age and gender. *J Cosmet Dermatol.* 2024;23:107-116. (<https://pubmed.ncbi.nlm.nih.gov/37616390/>)
- Cohen JL, Fagien S, Ogilvie P, et al. High patient satisfaction for up to 6 months with onabotulinumtoxinA treatment for upper facial lines. *Dermatol Surg.* 2022;48:1191-1197. (<https://pubmed.ncbi.nlm.nih.gov/36342250/>)
- Joseph JH, Maas C, Palm MD, et al. Safety, pharmacodynamic response, and treatment satisfaction with onabotulinumtoxinA 40 U, 60 U, and 80 U in subjects with moderate to severe dynamic glabellar lines. *Aesthet Surg J.* 2022;42:1318-1327. (<https://pubmed.ncbi.nlm.nih.gov/35704394/>)
- Cohen JL, Rivkin A, Dayan S, et al. Multimodal facial aesthetic treatment on the appearance of aging, social confidence, and psychological well-being: HARMONY study. *Aesthet Surg J.* 2022;42:NP115-NP124. (<https://pubmed.ncbi.nlm.nih.gov/33751048/>)
- Wu Y, Li C, Garcia J, Baradaran S. Patient-reported outcomes in Chinese subjects treated with onabotulinumtoxinA for crow's feet lines. *J Clin Aesthet Dermatol.* 2021;14:27-31. (<https://pubmed.ncbi.nlm.nih.gov/34976286/>)
- Keaney TC, Cavallini M, Leys C, et al. Efficacy, patient-reported outcomes, and safety in male subjects treated with onabotulinumtoxinA for improvement of moderate to severe horizontal forehead lines. *Dermatol Surg.* 2020;46:229-239. (<https://pubmed.ncbi.nlm.nih.gov/31343446/>)
- Palm MD, Few J, Patel T, et al. Efficacy, patient-reported outcomes, and safety for millennial subjects treated with onabotulinumtoxinA for moderate to severe horizontal forehead lines. *Dermatol Surg.* 2020;46:653-661. (<https://pubmed.ncbi.nlm.nih.gov/31625954/>)
- Ogilvie P, Rivkin AZ, Dayan S, et al. Pooled subject-reported outcomes from 2 Phase 3 studies of onabotulinumtoxinA for simultaneous treatment of forehead and glabellar lines. *Dermatol Surg.* 2020;46:950-957. (<https://pubmed.ncbi.nlm.nih.gov/31567611/>)
- Kawashima M, Harii K, Horiuchi Y, et al. Safety, efficacy, and patient satisfaction with onabotulinumtoxinA for the treatment of upper facial lines in Japanese subjects. *Dermatol Surg.* 2020;46:483-490. (<https://pubmed.ncbi.nlm.nih.gov/31517663/>)
- Rivkin AZ, Ogilvie P, Dayan S, Yoelin SG, Weichman BM, Garcia JK. OnabotulinumtoxinA for simultaneous treatment of upper facial lines: Subject-reported satisfaction and impact from a Phase 3 study. *Dermatol Surg.* 2020;46:50-60. (<https://pubmed.ncbi.nlm.nih.gov/30829771/>)
- Zhang X, Cai L, Yang M, Li F, Han X. Botulinum toxin to treat horizontal forehead lines: A refined injection pattern accommodating the lower frontalis. *Aesthet Surg J.* 2020;40:668-678. (<https://pubmed.ncbi.nlm.nih.gov/31250898/>)
- Ogilvie P, Rivkin AZ, Dayan S, Yoelin SG, Weichman BM, Garcia JK. OnabotulinumtoxinA for treatment of forehead and glabellar lines: Subject-reported satisfaction and impact from a Phase 3 double-blind study. *Dermatol Surg.* 2019;45:689-699. (<https://pubmed.ncbi.nlm.nih.gov/31034447/>)
- Montes JR, Ubale RV. Patient satisfaction and patients' family or significant other perceptions after onabotulinumtoxinA treatment: A prospective cross-sectional study. *Dermatol Surg.* 2019;45:1069-1079. (<https://pubmed.ncbi.nlm.nih.gov/30762713/>)
- Cohen JL, Swift A, Solish N, Fagien S, Glaser DA. OnabotulinumtoxinA and hyaluronic acid in facial wrinkles and folds: A prospective, open-label comparison. *Aesthet Surg J.* 2019;39:187-200. (<https://pubmed.ncbi.nlm.nih.gov/29762642/>)
- Wu Y, Wang G, Li C, Mao C, Lei X, Lee E. Safety and efficacy of onabotulinumtoxinA for treatment of crow's feet lines in Chinese subjects. *Plast Reconstr Surg Glob Open.* 2019;7:e2079. (<https://pubmed.ncbi.nlm.nih.gov/30859038/>)
- De Boulle K, Werschler WP, Gold MH, et al. Phase 3 study of onabotulinumtoxinA distributed between frontalis, glabellar complex, and lateral canthal areas for treatment of upper facial lines. *Dermatol Surg.* 2018;44:1437-1448. (<https://pubmed.ncbi.nlm.nih.gov/30096106/>)
- Wu Y, Lu Z, Xie Y, et al. OnabotulinumtoxinA treatment of moderate to severe glabellar lines in Chinese subjects after laser therapy: A prospective, open-label, noncomparative study. *J Cosmet Laser Ther.* 2018;20:278-286. (<https://pubmed.ncbi.nlm.nih.gov/29498553/>)
- Weinkle SH, Werschler WP, Teller CF, et al. Impact of comprehensive, minimally invasive, multimodal aesthetic treatment on satisfaction with facial appearance: The HARMONY study. *Aesthet Surg J.* 2018;38:540-556. (<https://pubmed.ncbi.nlm.nih.gov/29244069/>)
- Fagien S, Cohen JL, Coleman W, et al. Forehead line treatment with onabotulinumtoxinA in subjects with forehead and glabellar facial rhytids: A Phase 3 study. *Dermatol Surg.* 2017:43(Suppl 3):S274-S284. (<https://pubmed.ncbi.nlm.nih.gov/33065953/>)
- Harii K, Kawashima M, Furuyama N, Lei X, Hopfinger R, Lee E. OnabotulinumtoxinA (Botox) in the treatment of crow's feet lines in Japanese subjects. *Aesthetic Plast Surg.* 2017;41:1186-1197. (<https://pubmed.ncbi.nlm.nih.gov/28733805/>)
- Solish N, Rivers JK, Humphrey S, et al. Efficacy and safety of onabotulinumtoxinA treatment of forehead lines: A multicenter, randomized, dose-ranging controlled trial. *Dermatol Surg.* 2016;42:410-419. (<https://pubmed.ncbi.nlm.nih.gov/26863598/>)
- Rivers JK, Bertucci V, McGillivray W, et al. Subject satisfaction with onabotulinumtoxinA treatment of glabellar and lateral canthal lines using a new patient-reported outcome measure. *Dermatol Surg.* 2015;41:950-959. (<https://pubmed.ncbi.nlm.nih.gov/26218728/>)
- Dayan S, Coleman WP, Dover JS, et al. Effects of OnabotulinumtoxinA treatment for crow's feet lines on patient-reported outcomes. Dermatol Surg. 2015:41(Suppl 1):S67-S74. (<https://pubmed.ncbi.nlm.nih.gov/25548848/>)
- Moers-Carpi M, Carruthers J, Fagien S, et al. Efficacy and safety of onabotulinumtoxinA for treating crow's feet lines alone or in combination with glabellar lines: a multicenter, randomized, controlled trial. *Dermatol Surg.* 2015;41:102-112. (<https://pubmed.ncbi.nlm.nih.gov/25485803/>)
- Carruthers J, Rivkin A, Donofrio L, et al. A multicenter, randomized, double-blind, placebo-controlled study to evaluate the efficacy and safety of repeated onabotulinumtoxinA treatments in subjects with crow's feet lines and glabellar lines. *Dermatol Surg.* 2015;41:702-711. (<https://pubmed.ncbi.nlm.nih.gov/25993609/>)
- Carruthers A, Bruce S, De Coninck A, et al. Efficacy and safety of onabotulinumtoxina for the treatment of crows feet lines: a multicenter, randomized, controlled trial. *Dermatol Surg.* 2014;40:1181-1190. (<https://pubmed.ncbi.nlm.nih.gov/25347451/>)
- Gordin EA, Luginbuhl AL, Ortlip T, Heffelfinger RN, Krein H. Subcutaneous vs intramuscular botulinum toxin: split-face randomized study. *JAMA Facial Plast Surg.* 2014;16:193-198. (<https://pubmed.ncbi.nlm.nih.gov/24699554/>)
- Simão de Aquino M, Haddad A, Masako Ferreira L. Assessment of quality of life in patients who underwent minimally invasive cosmetic procedures. *Aesthetic Plast Surg.* 2013;37:497-503. (<https://pubmed.ncbi.nlm.nih.gov/23519872/>)
- Beer KR, Boyd C, Patel RK, Bowen B, James SP, Brin MF. Rapid onset of response and patient-reported outcomes after onabotulinumtoxinA treatment of moderate-to-severe glabellar lines. *J Drugs Dermatol.* 2011;10:39-44. (<https://pubmed.ncbi.nlm.nih.gov/21197522/>)
- Dailey RA, Philip A, Tardie G. Long-term treatment of glabellar rhytides using onabotulinumtoxinA. *Dermatol Surg.* 2011;37:918-928. (<https://pubmed.ncbi.nlm.nih.gov/21575099/>)
- Dayan SH, Arkins JP, Patel AB, Gal TJ. A double-blind, randomized, placebo-controlled health-outcomes survey of the effect of botulinum toxin type A injections on quality of life and self-esteem. *Dermatol Surg.* 2010;36(Suppl 4):2088-2097. (<https://pubmed.ncbi.nlm.nih.gov/21070456/>)
- Carruthers A, Carruthers J. A single-center dose-comparison study of botulinum neurotoxin type A in females with upper facial rhytids: assessing patients' perception of treatment outcomes. *J Drugs Dermatol.* 2009;8:924-929. (<https://pubmed.ncbi.nlm.nih.gov/19852121/>)
- Grimes PE, Shabazz D. A four-month randomized, double-blind evaluation of the efficacy of botulinum toxin type A for the treatment of glabellar lines in women with skin types V and VI. *Dermatol Surg.* 2009;35:429-435. (<https://pubmed.ncbi.nlm.nih.gov/19250310/>)
- Kawashima M, Harii K. An open-label, randomized, 64-week study repeating 10- and 20-U doses of botulinum toxin type A for treatment of glabellar lines in Japanese subjects. *Int J Dermatol.* 2009;48:768-776. (<https://pubmed.ncbi.nlm.nih.gov/19490208/>)
- de Boulle K. Patient satisfaction with different botulinum toxin type A formulations in the treatment of moderate to severe upper facial rhytids. *J Cosmet Laser Ther.* 2008;10:87-92. (<https://pubmed.ncbi.nlm.nih.gov/18569261/>)
- Harii K, Kawashima M. A double-blind, randomized, placebo-controlled, two-dose comparative study of botulinum toxin type A for treating glabellar lines in Japanese subjects. *Aesthetic Plast Surg.* 2008;32:724-730. (<https://pubmed.ncbi.nlm.nih.gov/18663516/>)
- Stotland MA, Kowalski JW, Ray BB. Patient-reported benefit and satisfaction with botulinum toxin type A treatment of moderate to severe glabellar rhytides: results from a prospective open-label study. *Plast Reconstr Surg.* 2007;120:1386-1393. (<https://pubmed.ncbi.nlm.nih.gov/17898617/>)
- Fagien S, Cox SE, Finn JC, Werschler WP, Kowalski JW. Patient-reported outcomes with botulinum toxin type A treatment of glabellar rhytids: a double-blind, randomized, placebo-controlled study. *Dermatol Surg.* 2007;33:S2-S9. (<https://pubmed.ncbi.nlm.nih.gov/17241410/>)
- Carruthers J, Carruthers A. Botulinum toxin type A treatment of multiple upper facial sites: patient-reported outcomes. *Dermatol Surg.* 2007;33:S10-S17. (<https://pubmed.ncbi.nlm.nih.gov/17241408/>)
- Beer KR. Comparative evaluation of the safety and efficacy of botulinum toxin type A and topical creams for treating moderate-to-severe glabellar rhytids. *Dermatol Surg.* 2006;32:184-197. (<https://pubmed.ncbi.nlm.nih.gov/16442037/>)
- Lowe P, Patnaik R, Lowe N. Comparison of two formulations of botulinum toxin type A for the treatment of glabellar lines: a double-blind, randomized study. *J Am Acad Dermatol.* 2006;55:975-980. (<https://pubmed.ncbi.nlm.nih.gov/17097394/>)
- Carruthers A, Carruthers J. Prospective, double-blind, randomized, parallel-group, dose-ranging study of botulinum toxin type A in men with glabellar rhytids. *Dermatol Surg.* 2005;31:1297-1303. (<https://pubmed.ncbi.nlm.nih.gov/16188182/>)
- Lowe NJ, Ascher B, Heckmann M, Kumar C, et al. Double-blind, randomized, placebo-controlled, dose-response study of the safety and efficacy of botulinum toxin type A in subjects with crow’s feet. *Dermatol Surg.* 2005;31:257-262. (<https://pubmed.ncbi.nlm.nih.gov/15841623/>)
- Lowe PL, Patnaik R, Lowe NJ. A comparison of two botulinum type A toxin preparations for the treatment of glabellar lines: double-blind, randomized, pilot study. *Dermatol Surg.* 2005;31:1651-1654. (<https://pubmed.ncbi.nlm.nih.gov/16336882/>)
- Carruthers A, Carruthers J, Said S. Dose-ranging study of botulinum toxin type A in the treatment of glabellar rhytids in females. *Dermatol Surg.* 2005;31:414-422. (<https://pubmed.ncbi.nlm.nih.gov/15871316/>)
- Patel MP, Talmor M, Nolan WB. Botox and collagen for glabellar furrows: Advantages of combination therapy. *Ann Plast Surg.* 2004;52:442-447. (<https://pubmed.ncbi.nlm.nih.gov/15096921/>)
- Cox SE, Finn JC, Stetler L, Mackowiak J, Kowalski JW. Development of the Facial Lines Treatment Satisfaction Questionnaire and initial results for botulinum toxin type A-treated patients. *Dermatol Surg.* 2003;29:444-449. (<https://pubmed.ncbi.nlm.nih.gov/12752509/>)

**AbobotulinumtoxinA [28]**

- Chadha P, Gerber PA, Hilton S, et al. Ready-to-use abobotulinumtoxinA solution versus powder botulinumtoxinA for treatment of glabellar lines: Investigators' and subjects' experience in a Phase IV study. *J Cosmet Dermatol.* 2024 May 28. Online ahead of print. (<https://pubmed.ncbi.nlm.nih.gov/38807515/>)
- Wu Y, Fang F, Lai W, et al. Efficacy and safety of abobotulinumtoxinA for the treatment of glabellar lines in Chinese patients: A pivotal, Phase 3, randomized, double-blind and open-label phase study. *Aesthetic Plast Surg.* 2023;47:351-364. (<https://pubmed.ncbi.nlm.nih.gov/36536093/>)
- Hilton S, Kestemont P, Sattler G, et al. Liquid abobotulinumtoxinA: Pooled data from two double-blind, randomized, placebo-controlled phase iii studies of glabellar line treatment. *Dermatol Surg.* 2022;48:1198-1202. (<https://pubmed.ncbi.nlm.nih.gov/36206385/>)
- Dayan S, Joseph J, Moradi A, et al. Subject satisfaction and psychological well-being with escalating abobotulinumtoxinA injection dose for the treatment of moderate to severe glabellar lines. *J Cosmet Dermatol.* 2022;21:2407-2416. (<https://pubmed.ncbi.nlm.nih.gov/35266281/>)
- Kestemont P, Hilton S, Andriopoulos B, et al. Long-term efficacy and safety of liquid abobotulinumtoxinA formulation for moderate-to-severe glabellar lines: A Phase III, double-blind, randomized, placebo-controlled and open-label study. *Aesthet Surg J.* 2022;42:301-313. (<https://pubmed.ncbi.nlm.nih.gov/34472596/>)
- Schlessinger J, Friedmann DP, Mayoral F, et al. AbobotulinumtoxinA treatment of glabellar lines using a new reconstitution and injection volume: Randomized, placebo-controlled data. *J Drugs Dermatol.* 2021;20:988-995. (<https://pubmed.ncbi.nlm.nih.gov/34491022/>)
- Smit R, Gubanova E, Kaufman J, et al. Patient satisfaction with abobotulinumtoxinA for aesthetic use in the upper face: A systematic literature review and post-hoc analysis of the APPEAL study. *J Clin Aesthet Dermatol.* 2021;14:E69-E88. (<https://pubmed.ncbi.nlm.nih.gov/34221231/>)
- Schlessinger J, Cohen JL, Shamban A, et al. A multicenter study to evaluate subject satisfaction with two treatments of abobotulinumtoxinA a year in the glabellar lines. *Dermatol Surg.* 2021;47:504-509. (<https://pubmed.ncbi.nlm.nih.gov/33165057/>)
- Cartier H, Hedén P, Delmar H, et al. Repeated full-face aesthetic combination treatment with abobotulinumtoxinA, hyaluronic acid filler, and skin-boosting hyaluronic acid after monotherapy with abobotulinumtoxinA or hyaluronic acid filler. *Dermatol Surg.* 2020;46:475-482. (<https://pubmed.ncbi.nlm.nih.gov/31592825/>)
- Ascher B, Rzany B, Kestemont P, et al. Significantly increased patient satisfaction following liquid formulation abobotulinumtoxinA treatment in glabellar lines: FACE-Q outcomes from a Phase 3 clinical trial. *Aesthet Surg J.* 2020;40:1000-1008. (<https://pubmed.ncbi.nlm.nih.gov/31550352/>)
- Cohen JL, Kaufman J, Peredo MI, Down R, Mashburn J. Assessment of psychological well-being after abobotulinumtoxinA treatment: A comparison of 2 reconstitution volumes. *Dermatol Surg.* 2020;46:289-292. (<https://pubmed.ncbi.nlm.nih.gov/30964785/>)
- Ascher B, Rzany B, Kestemont P, et al. Liquid formulation of abobotulinumtoxinA: A 6-month, Phase 3, double-blind, randomized, placebo-controlled study of a single treatment, ready-to-use toxin for moderate-to-severe glabellar lines. *Aesthet Surg J.* 2020;40:93-104. (<https://pubmed.ncbi.nlm.nih.gov/30893430/>)
- Hedén P, Hexsel D, Cartier H, et al. Effective and safe repeated full-face treatments with abobotulinumtoxinA, hyaluronic acid filler, and skin boosting hyaluronic acid. *J Drugs Dermatol.* 2019;18:682-689. (<https://pubmed.ncbi.nlm.nih.gov/31334927/>)
- Karbassi E, Nakhaee N, Zamanian M. The efficacy and complications of a new technique of Abobotulinum-toxin A (Dysport) injection in patients with glabellar lines. *J Cosmet Dermatol.* 2019;18:55-58. (<https://pubmed.ncbi.nlm.nih.gov/29569830/>)
- Kaufman J, Cohen JL, Peredo MI, Jonas B, Down R, Nogueira A. Clinical assessment of 2 licensed abobotulinumtoxinA injection volumes for the treatment of glabellar lines. *Dermatol Surg.* 2019;45:1274-1284. (<https://pubmed.ncbi.nlm.nih.gov/30893159/>)
- Hexsel D, Cartier H, Hedén P, et al. Efficacy, safety, and subject satisfaction after abobotulinumtoxinA treatment of upper facial lines. *Dermatol Surg.* 2018;44:1555-1564. (<https://pubmed.ncbi.nlm.nih.gov/30204739/>)
- Gubanova E, Haddad Tabet M, Bergerova Y, et al. Assessment of subject and physician satisfaction after long-term treatment of glabellar lines with abobotulinumtoxinA (Dysport®/Azzalure®): Primary results of the APPEAL noninterventional study. *Aesthetic Plast Surg.* 2018;42:1672-1680. (<https://pubmed.ncbi.nlm.nih.gov/30120518/>)
- Elridy AS, Zaki RGE, Elshinawy RF. Comparison of the clinical efficacy of abobotulinumtoxin A (abo) and onabotulinumtoxin A (ona) in the treatment of crow's feet wrinkles: A split-face study. *Semin Ophthalmol.* 2018;33:739-747. (<https://pubmed.ncbi.nlm.nih.gov/29278961/>)
- Rostedt Punga A, Alimohammadi M, Fagrell D, Nyberg F, Rees D, Wong C. A randomized, comparative study to evaluate efficacy and safety of two injection volumes of abobotulinumtoxinA in treatment of glabellar Lines. *Dermatol Surg.* 2016;42:967-976. (<https://pubmed.ncbi.nlm.nih.gov/27399956/>)
- Molina B, David M, Jain R, et al. Patient satisfaction and efficacy of full-facial rejuvenation using a combination of botulinum toxin type A and hyaluronic acid filler. *Dermatol Surg.* 2015:41(Suppl 1):S325-S332. (<https://pubmed.ncbi.nlm.nih.gov/26618460/>)
- Molina B, Grangier Y, Mole B, et al. Patient satisfaction after the treatment of glabellar lines with Botulinum toxin type A (Speywood Unit): a multi-centre European observational study. *J Eur Acad Dermatol Venereol.* 2015;29:1382-1388. (<https://pubmed.ncbi.nlm.nih.gov/25495499/>)
- Beer KR, Julius H, Dunn M, Wilson F. Remodeling of periorbital, temporal, glabellar, and crow's feet areas with hyaluronic acid and botulinum toxin. *J Cosmet Dermatol.* 2014;13:143-150. (<https://pubmed.ncbi.nlm.nih.gov/24910278/>)
- Hexsel D, Brum C, Porto MD, et al. Quality of life and satisfaction of patients after full-face injections of abobotulinum toxin type A: A randomized, phase IV clinical trial. *J Drugs Dermatol.* 2013;12:1363-1367. (<https://pubmed.ncbi.nlm.nih.gov/24301237/>)
- Kassir R, Kolluru A, Kassir M. Triple-blind, prospective, internally controlled comparative study between abobotulinumtoxinA and onabotulinumtoxinA for the treatment of facial rhytids. *Dermatol Ther (Heidelb).* 2013;3:179-189. (<https://pubmed.ncbi.nlm.nih.gov/24318416/>)
- Ascher B, Rzany BJ, Grover R. Efficacy and safety of botulinum toxin type A in the treatment of lateral crow's feet: double-blind, placebo-controlled, dose-ranging study. *Dermatol Surg.* 2009;35:1478-1486. (<https://pubmed.ncbi.nlm.nih.gov/19686365/>)
- Rzany B, Ascher B, Fratila A, Monheit GD, Talarico S, Sterry W. Efficacy and safety of 3- and 5-injection patterns (30 and 50 U) of botulinum toxin A (Dysport) for the treatment of wrinkles in the glabella and the central forehead region. *Arch Dermatol.* 2006;142:320-326. (<https://pubmed.ncbi.nlm.nih.gov/16549707/>)
- Ascher B, Zakine B, Kestemont P, Baspeyras M, et al. Botulinum toxin A in the treatment of glabellar lines: scheduling the next injection. *Aesthet Surg J.* 2005;25:365-375. (<https://pubmed.ncbi.nlm.nih.gov/19338833/>)
- Ascher B, Zakine B, Kestemont P, Baspeyras M, Bougara A, Santini J. A multicenter, randomized, double-blind, placebo-controlled study of efficacy and safety of 3 doses of botulinum toxin A in the treatment of glabellar lines. *J Am Acad Dermatol.* 2004;51:223-233. (<https://pubmed.ncbi.nlm.nih.gov/15280841/>)

**IncobotulinumtoxinA [9]**

- Trindade de Almeida A, de Sanctis Pecora C, Marques ER, Contin L, Trindade de Almeida C, da Cunha AL. Assessment of the efficacy and durability of incobotulinumtoxinA in the treatment of the upper face in adult women. *Dermatol Ther (Heidelb).* 2024 Jul 2. Online ahead of print. (<https://pubmed.ncbi.nlm.nih.gov/38954382/>)
- Barbarino SC, van Loghem JAJ, Burgess CM, Corduff N. Evaluating the effect of incobotulinumtoxin A for glabellar, forehead, and crow's feet lines using a high dilution. *J Clin Aesthet Dermatol.* 2021;14:34-40. (<https://pubmed.ncbi.nlm.nih.gov/34840655/>)
- Fischer T, Sattler G, Prager W, et al. Safety, tolerability, and efficacy of repeat-dose injections of incobotulinumtoxinA in the treatment of upper facial lines: Results from a prospective, open-label, Phase III study. *J Drugs Dermatol.* 2020;19:461-469. (<https://pubmed.ncbi.nlm.nih.gov/32484631/>)
- Chao YYY, Tseng FW, Yang YL, Chen YH, Hsu NJ, Chang LY. IncobotulinumtoxinA for the treatment of glabellar frown lines: A prospective, multicenter, single-arm study in Taiwan. *J Clin Aesthet Dermatol.* 2019;12:E53-E57. (<https://pubmed.ncbi.nlm.nih.gov/32038766/>)
- Lim JTE, Loh DKT, Soh K, Sunga O. Efficacy and patient satisfaction with incobotulinumtoxinA for the treatment of glabellar frown lines. *Singapore Med J.* 2017;58:606-609. (<https://pubmed.ncbi.nlm.nih.gov/27357317/>)
- Streker M, Luebberding S, Krueger N, Harrington L, Kerscher M. Patient-reported outcomes after incobotulinumtoxinA treatment for upper facial wrinkles. *Dermatol Surg.* 2015;41(Suppl 1):S29-S38. (<https://pubmed.ncbi.nlm.nih.gov/25548843/>)
- Kane MAC, Gold MH, Coleman WP, et al. A randomized, double-blind trial to investigate the equivalence of incobotulinumtoxinA and onabotulinumtoxinA for glabellar frown lines. *Dermatol Surg.* 2015;41:1310-1319. (<https://pubmed.ncbi.nlm.nih.gov/26509943/>)
- Jandhyala R. Impact of botulinum toxin A on the quality of life of subjects following treatment of facial lines. *J Clin Aesthet Dermatol.* 2013;6:41-45. (<https://pubmed.ncbi.nlm.nih.gov/24062873/>)
- Prager W, Bee EK, Havermann I, Zschocke I. Onset, longevity, and patient satisfaction with incobotulinumtoxinA for the treatment of glabellar frown lines: a single-arm, prospective clinical study. *Clin Interv Aging.* 2013:8:449-456. (<https://pubmed.ncbi.nlm.nih.gov/23650444/>)

**PrabotulinumtoxinA [11]**

- Taylor-Barnes K. Real-world patient experience with prabotulinumtoxinA in the United Kingdom: A single-center survey and analysis of 254 patients. *Aesthet Surg J Open Forum.* 2024;6:ojae013. (<https://pubmed.ncbi.nlm.nih.gov/38828475/>)
- Cox SE, Ascher B, Avelar RL, et al. PrabotulinumtoxinA for the treatment of glabellar lines in adults, 65 years of age and older: The fourth in a series of post hoc analyses of the phase III clinical study data. *J Cosmet Dermatol.* 2023;22:1745-1756. (<https://pubmed.ncbi.nlm.nih.gov/37102990/>)
- Lee SK, Kim MS, Kwon SH, Chung BY, Han SH, Kim HJ. Efficacy, safety, and subject satisfaction of prabotulinumtoxinA for moderate-to-severe crow's feet: A Phase IV, multicenter, double-blind, randomized, placebo-controlled trial. *J Clin Med.* 2023;12:6326. (<https://pubmed.ncbi.nlm.nih.gov/37834970/>)
- Vasile G, Green C, Bhatti H, et al. OnabotulinumtoxinA versus prabotulinumtoxinA-xvfs: A randomized, triple-blind, split-face study on the time to onset, rhytid appearance, and patient satisfaction in forehead and glabellar lines. *J Clin Aesthet Dermatol.* 2023;16:47-49. (<https://pubmed.ncbi.nlm.nih.gov/37288279/>)
- Ogilvie P, Jones DH, Avelar RL, Jonker A, Monroe R, Carruthers J. PrabotulinumtoxinA for treatment of millennials with moderate to severe glabellar lines: Post hoc analyses of the Phase III clinical study data. *Dermatol Surg.* 2022;48:664-669. (<https://pubmed.ncbi.nlm.nih.gov/35616616/>)
- Solish N, Ascher B, Avelar RL, et al. PrabotulinumtoxinA vs onabotulinumtoxinA for the treatment of adult males with moderate to severe glabellar lines: Post-hoc analyses of the phase III clinical study data. *Aesthet Surg J.* 2022;42:1460-1469. (<https://pubmed.ncbi.nlm.nih.gov/35922149/>)
- Taylor SC, Grimes PE, Joseph JH, Jonker A, Avelar RL. PrabotulinumtoxinA for the treatment of moderate-to-severe glabellar lines in adult patients with skin of color: Post hoc analyses of the US Phase III clinical study data. *Dermatol Surg.* 2021;47:516-521. (<https://pubmed.ncbi.nlm.nih.gov/33165078/>)
- Rzany BJ, Ascher B, Avelar RL, et al. A multicenter, randomized, double-blind, placebo-controlled, single-dose, phase III, non-inferiority study comparing prabotulinumtoxinA and onabotulinumtoxinA for the treatment of moderate to severe glabellar lines in adult patients. *Aesthet Surg J.* 2020;40:413-429. (<https://pubmed.ncbi.nlm.nih.gov/30951166/>)
- Beer KR, Shamban AT, Avelar RL, Gross JE, Jonker A. Efficacy and safety of prabotulinumtoxinA for the treatment of glabellar lines in adult subjects: Results From 2 identical Phase III studies. *Dermatol Surg.* 2019;45:1381-1393. (<https://pubmed.ncbi.nlm.nih.gov/30893162/>)
- Cheon HI, Jung N, Won CH, Kim BJ, Lee YW. Efficacy and safety of prabotulinumtoxin A and onabotulinumtoxin A for crow's feet: A Phase 3, multicenter, randomized, double-blind, split-face study. *Dermatol Surg.* 2019;45:1610-1619. (<https://pubmed.ncbi.nlm.nih.gov/30893169/>)
- Won CH, Kim HK, Kim BJ, et al. Comparative trial of a novel botulinum neurotoxin type A versus onabotulinumtoxinA in the treatment of glabellar lines: a multicenter, randomized, double-blind, active-controlled study. *Int J Dermatol.* 2015;54:227-234. (<https://pubmed.ncbi.nlm.nih.gov/25311357/>)

**DaxibotulinumtoxinA [3]**

- Dover JS, Humphrey SD, Lorenc ZP, et al. Treatment of upper facial lines with daxibotulinumtoxinA for injection: Results from an open-label Phase 2 study. *Dermatol Surg.* 2023;49:60-65. (<https://pubmed.ncbi.nlm.nih.gov/36533798/>)
- Bertucci V, Solish N, Kaufman-Janette J, et al. DaxibotulinumtoxinA for Injection has a prolonged duration of response in the treatment of glabellar lines: Pooled data from two multicenter, randomized, double-blind, placebo-controlled, phase 3 studies (SAKURA 1 and SAKURA 2). *J Am Acad Dermatol.* 2020;82:838-845. (<https://pubmed.ncbi.nlm.nih.gov/31791824/>)
- Carruthers JD, Fagien S, Joseph JH, et al. DaxibotulinumtoxinA for injection for the treatment of glabellar lines: Results from each of two multicenter, randomized, double-blind, placebo-controlled, Phase 3 studies (SAKURA 1 and SAKURA 2). *Plast Reconstr Surg.* 2020;145:45-58. (<https://pubmed.ncbi.nlm.nih.gov/31609882/>)

**LetibotulinumtoxinA [3]**

- Cox SE, Kaufman-Janette J, Cohen JL, et al. LetibotulinumtoxinA attenuates the psychological burden of glabellar lines and is associated with high subject satisfaction in Phase 3 clinical trials. *Dermatol Surg.* 2024;50:535-541. (<https://pubmed.ncbi.nlm.nih.gov/38470985/>)
- Yoo KH, Park SJ, Han HS, Won CH, Lee YW, Kim BJ. Randomized, double-blind, active-controlled, multicentre, phase III clinical trial with two stages to assess the safety and efficacy of letibotulinum toxin A vs. onabotulinum toxin A for subjects with moderate to severe crow's feet. *J Eur Acad Dermatol Venereol.* 2021;35:1587-1594. (<https://pubmed.ncbi.nlm.nih.gov/33721365/>)
- Kim BJ, Kwon HH, Park SY, et al. Double-blind, randomized non-inferiority trial of a novel botulinum toxin A processed from the strain CBFC26, compared with onabotulinumtoxin A in the treatment of glabellar lines. *J Eur Acad Dermatol Venereol.* 2014;28:1761-1767. (<https://pubmed.ncbi.nlm.nih.gov/24593323/>)

**Various botulinum toxins [1]**

- Chang BL, Wilson AJ, Taglienti AJ, Chang CS, Folsom N, Percec I. Patient perceived benefit in facial aesthetic procedures: FACE-Q as a tool to study botulinum toxin injection outcomes. *Aesthet Surg J.* 2016;36:810-820. (<https://pubmed.ncbi.nlm.nih.gov/26780945/>)
